# Supplementary figures and images for: Histological Analysis of a Mouse Model of the 22q11.2 Microdeletion Syndrome
Source: Biomolecules. 2023 Apr 27;13(5):763. doi: 10.3390/biom13050763 (PMC10216591; doi:10.3390/biom13050763)

## Supplementary Figure S1

Dendritic spine density of AAV-labeled neurons

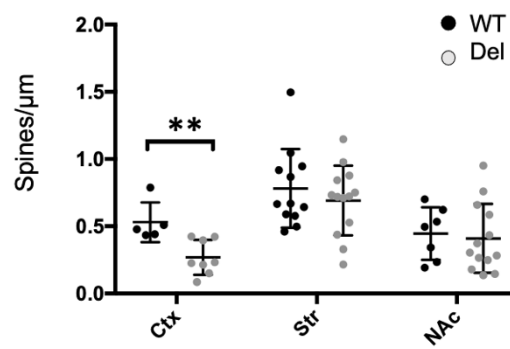

Supplement: Supplementary file 1 [file biomolecules-13-00763-s001.zip › biomolecules-2363633-supplementary.pdf]
